# Why People With Parkinson's Disease Experience Near‐Drowning—and How to Prevent It

**DOI:** 10.1002/mdc3.12989

**Published:** 2020-06-02

**Authors:** Anouk Tosserams, Maarten J. Nijkrake, Nicoline B.M. Voet, Bastiaan R. Bloem, Jorik Nonnekes

**Affiliations:** ^1^ Department of Neurology, Center of Expertise for Parkinson & Movement Disorders Radboud University Medical Centre; Donders Institute for Brain, Cognition and Behaviour Nijmegen The Netherlands; ^2^ Department of Rehabilitation, Center of Expertise for Parkinson & Movement Disorders Radboud University Medical Centre; Donders Institute for Brain, Cognition and Behaviour Nijmegen The Netherlands; ^3^ Klimmendaal, Rehabilitation Center Arnhem The Netherlands; ^4^ Department of Rehabilitation Sint Maartenskliniek Nijmegen The Netherlands

**Keywords:** Parkinson's disease, swimming, rehabilitation, compensation strategies

## Abstract

View Supplementary Video S1

View Supplementary Video S2

View Supplementary Video S3

The merits of exercise for persons with Parkinson's disease (PD) are increasingly recognized.^1^ Swimming is a common way to stay active for persons with PD. However, recent work shows that swimming in PD is not without risks: A survey conducted among 309 persons with PD indicated that 87.7% had noticed a deterioration in their swimming capacity, and 49.1% reported near‐drowning experiences.^2^ In this video‐illustrated report, we provide a detailed analysis of how swimming has become affected in an experienced swimmer with PD. We also highlight how both pharmacological and nonpharmacological interventions improved his swimming capacity, which may help to prevent near‐drowning or actual drowning.

For 3 years, a 56‐year‐old proficient swimmer experienced a decline in his swimming abilities. Specifically, he noticed his left leg was progressively lagging during the crawl. Over the course of 2 years, this escalated until he could no longer swim independently, because of a drowning risk. By then, he had also developed a mild resting tremor of his left hand, hyposmia, and hypokinetic gait. He was diagnosed with PD (H & Y stage II). Video 1 shows his swimming pattern before the start of dopaminergic treatment. Bradykinesia and poor interlimb coordination result in decreased propulsive force and failure to maintain a horizontal body position.^3^ Levodopa/carbidopa 187.5 mg three times daily improved his swimming abilities (Video 2). The application of compensation strategies (using swim fins, using the legs only, and internal cueing: counting and making a stroke at every count) was also beneficial, over and above the effect of dopaminergic therapy alone (Video 3).

The importance of this observation is three fold. First, we illustrate that near‐drowning in persons with PD is probably caused by both bradykinesia and impaired interlimb coordination. The underlying mechanism likely involves impaired corticostriatal input, as suggested by a recent report of PD patients losing their swimming ability after STN‐DBS.^4^ Second, we show that dopaminergic medication improves swimming by reducing bradykinesia, despite it having no effect on the impaired interlimb coordination. Third, we illustrate that compensation strategies—essential in managing gait impairments in PD—can also improve swimming. The underlying mechanisms likely depend on the type of strategy. When using swim fins, a greater propulsive force is generated given that more water is displaced per stroke, facilitating the maintenance of a horizontal body position in the water. Fins may also work as a tactile cue: Increased water resistance to the feet could trigger patients to make larger leg strokes. Another solution to improve swimming is to bypass the necessity of interlimb coordination, by using the legs only. By applying this strategy, leg movement frequency is no longer “plagued” by the typically slower arm movements. Indeed, when the patient was asked to hold a floating board with his arms, his leg stroke efficiency increased spectacularly in terms of both frequency and force. Internal cueing presumably made swimming more goal directed, bypassing defective corticostriatal loops.^5^

Regardless of the exact underlying mechanisms, the clinical implications are evident. Persons with PD should be well informed about the potential drowning hazard. And, importantly, swimming capacity can be improved through a complementary approach of pharmacological and nonpharmacological interventions.

## Author Roles

(1) Research Project: A. Conception, B. Organization, C. Execution; (2) Manuscript: A. Writing of the First Draft, B. Review and Critique.

A.T.: 1A, 1B, 1C, 2A, 2B

M.N.: 1B, 2B

N.V.: 1B, 2B

B.B.: 1A, 1B, 2B

J.N.: 1A, 1B, 2B

## Disclosures

**Ethical Compliance Statement:** This study was performed in accordance with the guidelines proposed in the Declaration of Helsinki. IRB approval was not necessary for this work. Written consent was obtained from the patient involved. The corresponding author (A.T.) confirms that authorization signed by the depicted patient has been obtained in compliance with the applicable regulations regarding patient authorizations relating to the use or disclosure of protected health information. The 3 nonpatients visible in the video have all agreed that the figure with their image might be published. We confirm that we have read the Journal's position on issues involved in ethical publication and affirm that this work is consistent with those guidelines.

**Funding Sources and Conflicts of Interest:** The authors report no sources of funding and no conflicts of interest.

**Financial Disclosures for previous 12 months:** J.N. was supported by ZonMw and the Michael J. Fox Foundation. B.B. has received honoraria from serving on the scientific advisory board for AbbVie, Biogen, UCB, and Walk with Path; has received fees for speaking at conferences from AbbVie, Zambon, Roche, GE Healthcare, and Bial; and has received research support from The Netherlands Organisation for Scientific Research, the Michael J. Fox Foundation, UCB, AbbVie, the Stichting Parkinson Fonds, the Hersenstichting Nederland, the Parkinson Foundation, Verily Life Sciences, Horizon 2020, the Topsector Life Sciences and Health, and the Parkinson Vereniging.

## Supporting information


**Video 1.** Swimming before the start of dopaminergic treatment (l‐dopa naïve). Marked bradykinesia—characterized by slowness, reduced movement amplitude, and sequence effect—is present during both crawl and breaststroke. Bradykinesia is most pronounced on the left side. Additionally, interlimb coordination is affected during the crawl: Normally, the frequency of leg movements during the crawl is higher than the frequency of arm movements, but the arm‐to‐leg movement ratio is 1/1 in this patient.

**Video 2.** Swimming in the *on* dopaminergic state. Bradykinesia is reduced after dopaminergic treatment. However, the impaired interlimb coordination does not seem to be affected by l‐dopa: The arm‐to‐leg movement ratio remains 1/1.

**Video 3.** Swimming in the *on* dopaminergic state, with additional use of compensation strategies. A more effective leg stroke (in terms of both frequency and force) is achieved by using swim fins, as well as by holding a floating board. Use of internal cueing (counting and making a leg stroke at every count) appears to improve the amplitude of the stroke during both crawl and breaststroke, but does not improve leg stroke frequency.
